# Prevalence and correlates of suicidal behaviour in adolescents with bipolar disorder

**DOI:** 10.1192/j.eurpsy.2021.102

**Published:** 2021-08-13

**Authors:** S. Romero, I. Mendez, S. Lera, P. Santamarina

**Affiliations:** 1 Department Of Child And Adolescent Psychiatry And Psychology, Institute Clinic of Neurosciences, Hospital Clinic of Barcelona, Barcelona, Spain; 2 Cibersam, Instituto Carlos III, Madrid, Spain

**Keywords:** bipolar disorder, adolescents, suicidal behavior

## Abstract

**Objective:**

To examine the prevalence and correlates of suicidal behavior among adolescents with bipolar disorder (BD).

**Methods:**

47 adolescents, ages 12 to 19 years (15.8 ± 2), meeting DSM-5 criteria for BD-I (n=40) and BD-II (n=7) were assessed using the KSADS-PL and tested with a battery of tests measuring mood, psychotic symptoms, life events and functioning. History of suicidal attempts (SA) was ascertained using the K-SADS-PL.**Results:** One third (n=15, 32%) of the BD sample had a lifetime history of SA. There were no differences in socio-demographics factors between SA versus non- SA. BD adolescents with lifetime SA, were more likely to have lower weight at birth, a lifetime history of comorbid eating disorder, non-suicidal self-injurious behavior, 2nd degree family history of suicide attempt, and more stressful life events as compared with non-attempters. Adolescents with lifetime history of SA also showed statistically significant higher scores in depression, suicidal ideation and anxiety as compared with BD adolescents without lifetime SA. Logistic regression analysis found that the most robust correlates of SA in adolescents with BD were having 2nd degree family history of SA, the interaction of self-injury behavior and comorbid eating disorder and increased number of life events. **Limitations:** Retrospective data. Small sample size. Since this is a cross-sectional study, no inferences regarding causality can be made. **Conclusion:** One third of the adolescents with BD have attempted suicide. These results are in agreement with previous studies. History of SA in adolescents with BD is strongly associated with family history of suicidal behavior, lifetime self-injury behavior with comorbid eating disorder and increased number of stressful life events.

**Disclosure:**

No significant relationships.

